# A Case of a Young Pregnant Patient With Congenital Portal Vein Stenosis, Portal Hypertension, and Liver Cirrhosis

**DOI:** 10.7759/cureus.92062

**Published:** 2025-09-11

**Authors:** Fatimah A Alzain, Raheeq J Alolaian, Areej J Alolayan, Anoar J Alolaian, Yasmeen H Albadrani

**Affiliations:** 1 Obstetrics and Gynecology, Qatif Central Hospital, Qatif, SAU

**Keywords:** congenital portal vein stenosis, liver cirrhosis, obstetrics and gynecology, portal hypertension, pregnancy

## Abstract

Congenital portal vein stenosis is a narrowing of the portal vein that may lead to liver cirrhosis. It is an abnormality that is usually discovered accidentally following investigation of a metabolic disturbance or intra-abdominal tumors in children or adults. Here, we describe the case of a 23-year-old pregnant woman with a medical history of congenital portal vein stenosis complicated by liver cirrhosis and portal vein hypertension. The patient presented at 36 weeks of gestation with generalized body edema. Following a normal vaginal delivery, significant postpartum hemorrhage occurred, which required multidisciplinary team involvement and intensive care unit admission. Ultimately, a clinical and hemodynamic response was noted. This case of a young female with these conditions illustrates the complexities involved in managing such pregnancies. Congenital vein stenosis is a rare condition that may be exacerbated by the physiological changes that already occur during pregnancy. Fetal and maternal complication risk increases, including intrauterine growth restriction, spontaneous abortions, and maternal death following hemodynamic compromise. Hence, advanced measures and clinical anticipation of complications in such patients will significantly enhance both maternal and fetal outcomes.

## Introduction

Congenital portal vein stenosis is a narrowing of the portal vein that subsequently may lead to liver cirrhosis. It is an abnormality that is usually discovered accidentally following investigation of a metabolic disturbance or intra-abdominal tumors in children or adults [1]. Congenital portal vein stenosis occurs in about 1 in every 30,000 births [2].

Complicated congenital portal vein stenosis is a rare condition to be encountered and managed during pregnancy, affecting nearly 3% of pregnancies, which requires a multidisciplinary approach [3]. Pregnancy in the presence of cirrhosis is defined as permanent scarring of the liver [4].

The physiological changes that occur during pregnancy include increased blood volume and changes in hemodynamics. Moreover, the cardiovascular system undergoes dramatic changes. More recent data using Doppler ultrasound have shown an increase in portal vein flow by 50% in late pregnancy with a corresponding increase in total liver blood flow [4], which may exacerbate the already compromised liver function in affected women.

Additionally, portal hypertension can lead to considerable maternal complications, with mortality rates among affected women ranging between 2% and 18% [5]. Past evidence indicates that 20% of pregnant women with cirrhosis in developed nations may die during their pregnancy. Nonetheless, recent research shows improved results, with mortality rates <2% [6].

Multidisciplinary team interventions are required in such cases due to a lack of definitive measures in the management of complicated congenital portal vein stenosis in pregnant patients and the insufficiency of cases reported. This case report aims to emphasize the significance of care provided to pregnant patients with congenital vein stenosis, highlighting the anticipated risks in these patients. In this report, we present the case of a young primigravida patient with a diagnosis of congenital portal vein stenosis complicated with portal hypertension and liver cirrhosis, who had an eventful hospital stay.

## Case presentation

A 23-year-old pregnant female, primigravida, was referred to our hospital at 36 weeks and six days of gestation, as determined by the first-trimester ultrasound, which aligned with the patient’s last menstrual period. She presented with complaints of generalized body edema. Her medical history was significant for liver cirrhosis secondary to congenital portal vein stenosis/thrombosis with portal hypertension, diagnosed incidentally at the age of 11 after complaining of epistaxis and petechial rash. She had a history of pancytopenia, previously requiring platelet transfusion, and occasional blood transfusions due to low hemoglobin. In May 2024, the patient underwent an esophagogastroduodenoscopy (EGD). Although she had a history of esophageal varices the previous year, the recent EGD showed no esophageal or gastric varices, and only a few non-ulcerative gastric erosions were identified. Additionally, the patient had no history of hepatic encephalopathy or spontaneous bacterial peritonitis. The patient was a nonsmoker with no history of alcohol use or previous surgeries.

**Figure 1:**
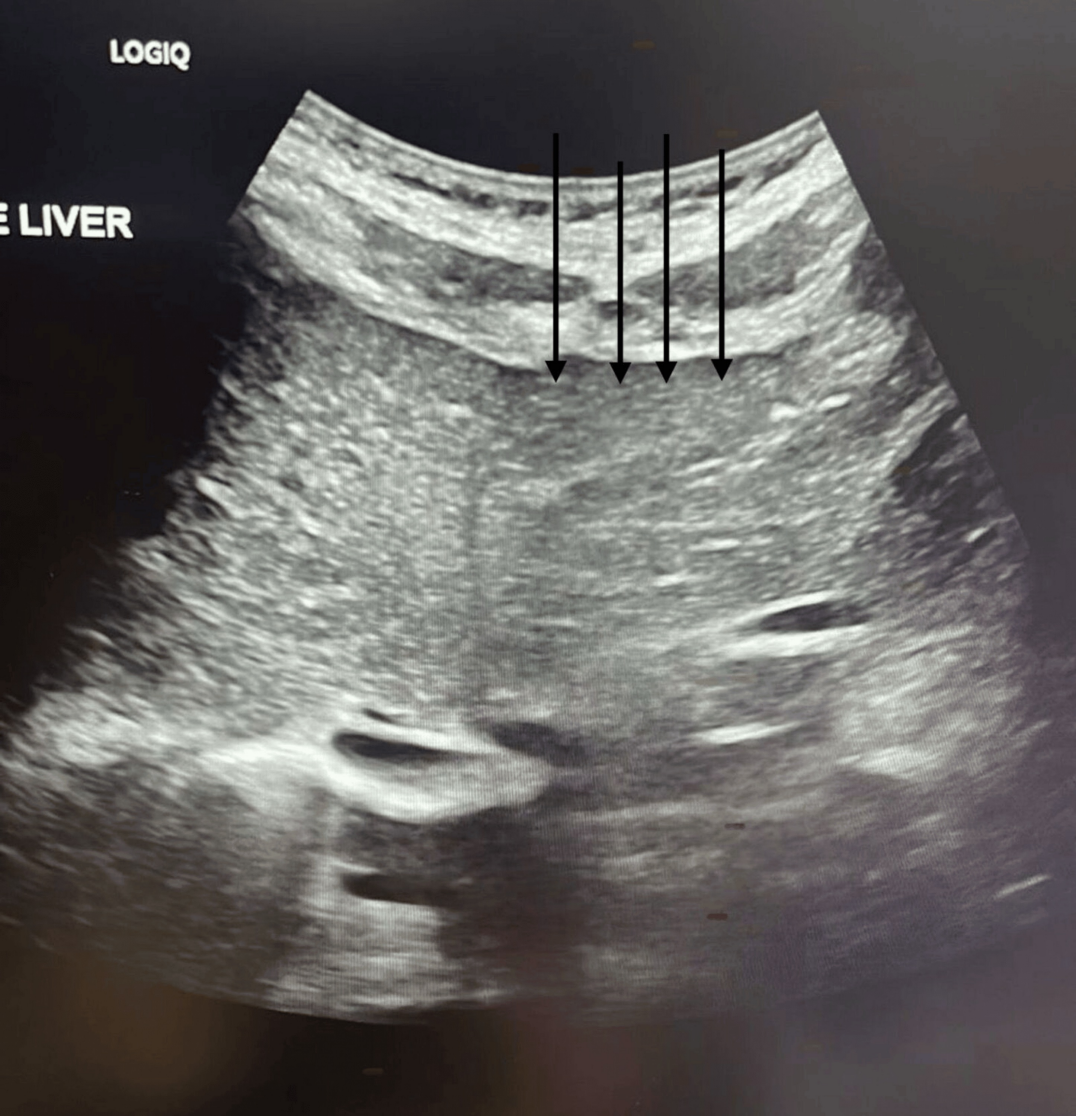
Ultrasound of the liver demonstrating cirrhotic changes. Nodular liver surface can be noted with no detectable hepatic focal lesion.

**Figure 2:**
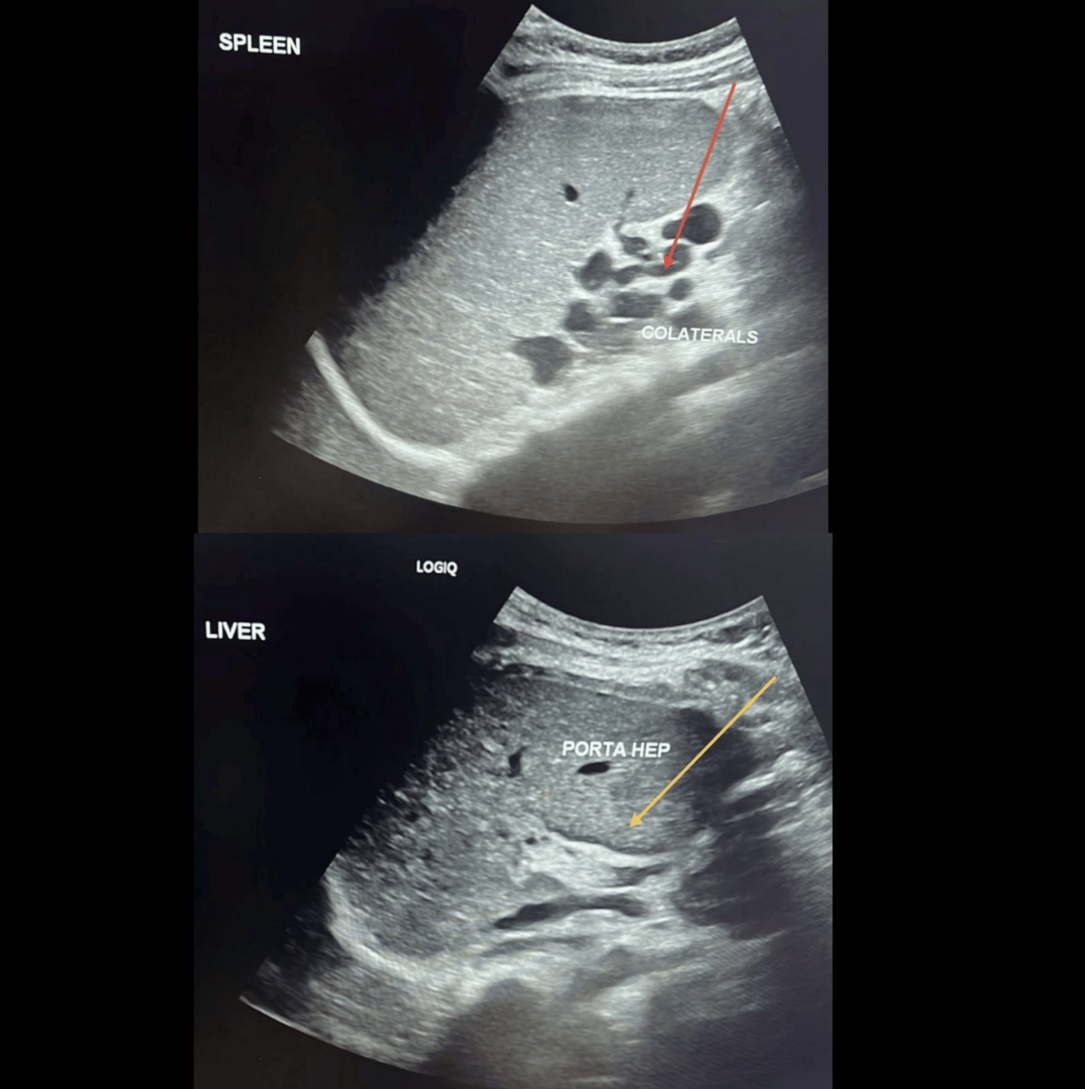
Ultrasound of the spleen illustrating a splenomegaly with multiple vascular collaterals at the splenic hilum (red arrow) and porta hepatis (yellow arrow), suggestive of portal hypertension.

On examination, the patient appeared stable with a normal fetal heart rate. Her vital signs were within normal limits: temperature, afebrile; blood pressure, 120/64 mmHg; pulse, 89 beats/minute; and oxygen saturation (SpO₂), 99% on room air. Obstetric ultrasound confirmed a single viable fetus in cephalic presentation, with an estimated fetal weight of 2.6 kg. Table 1 presents the laboratory investigations performed for the patient. An echocardiogram showed normal left and right ventricular size and function, with an ejection fraction of >55%, and no significant valve abnormalities, ruling out cardiac causes for her edema. The last liver ultrasound revealed a cirrhotic liver with signs of portal hypertension and no focal hepatic lesions (Figure 1). The unchanged appearance of the chronic portal vein thrombosis showed multiple porta-hepatis collaterals. Splenomegaly with multiple vascular collaterals at the porta-hepatis was suggestive of portal hypertension (Figure 2).

**Table 1:** Laboratory investigations. Note: Reference ranges are from the laboratories of the authors’ institutions.

Variable	Laboratory results	Reference ranges
Hemoglobin	10.9	12–15 g/dL
White blood cells	4.67	4–10 × 10^3^/µL
Platelets	85.4	150–430 × 10^3^/µL
Prothrombin time	17.2	11.5–15.5 seconds
International normalized ratio	1.28	0.85–1.15
D-dimer	1.23	0–0.5 µg/mL
Fibrinogen	1.59	2.55–3.65 g/L
Total protein	50	64–82 g/L
Albumin	25	35–52 g/L
Bilirubin (conjugated)	69	0–5.13 µmol/L
Bilirubin (total)	81	0–20.52 µmol/L
Sodium	142	135–153 mmol/L
Potassium	4.38	3.5–5.5 mmol/L
Urea	2.7	1.66–8.33 mmol/L
Creatinine	71	44–88 µmol/L

The patient’s condition required a multidisciplinary team approach for optimal management. The gastroenterology department agreed to proceed with normal vaginal delivery, with close observation for decompensation signs, while the hematology department assessed her thrombocytopenia and coagulopathy as a secondary complication of her liver disease, with no current indication for platelet transfusion. The intensive care unit team was consulted for post-delivery observation, and the blood bank prepared units of fresh frozen plasma (FFP), packed red blood cells, and platelets in anticipation of possible postpartum hemorrhage.

The patient underwent vaginal delivery assisted by vacuum extraction after failure of descent with initial pulls. She delivered a healthy male infant weighing 2.33 kg with Apgar scores of 7 and 10. Active management of the third stage of labor was performed with oxytocin and tranexamic acid. However, she developed considerable atonic postpartum hemorrhage with a drop in hemoglobin from 10 g/dL to 6 g/dL, necessitating the transfusion of four units of FFP and six units of platelets. Factor VIIa was administered as per hematology recommendations, which aligns with emergency protocols for cirrhosis-related coagulopathy [3]. Balloon tamponade was performed to control the bleeding. The hemorrhage was attributed to coagulopathy, and the patient responded well to both uterotonics (oxytocin and tranexamic acid), balloon tamponade, and blood transfusion, without the need for surgical intervention.

Following delivery, the patient was transferred to the intensive care unit for close monitoring. Postpartum management included continuous monitoring of vital signs, per-vaginal bleeding, and laboratory parameters. The patient was initiated on antibiotics to mitigate the risk of postpartum infections, particularly in the context of her cirrhosis-related immunocompromised state. Thromboprophylaxis was introduced postoperatively, and unfractionated heparin 5,000 IU twice a day was initiated 24 hours postpartum after confirmation of hemostasis.

The patient responded well to the multidisciplinary management approach. She remained hemodynamically stable with no further complications. The patient was subsequently discharged home on day five postpartum in a stable condition with follow-up arranged.

## Discussion

Despite earlier beliefs, advancements in medicine indicate that pregnancy is not contraindicated for women with liver disease, with the pregnancy outcomes depending on the severity of maternal liver disease [3]. The Model for End-stage Liver Disease (MELD) has proven effective in forecasting the likelihood of maternal liver disease risk during pregnancy [3]. The MELD score, which includes serum creatinine, bilirubin, and international normalized ratio, was originally developed to estimate the mortality risk for patients with cirrhosis undergoing procedures. Women who have a MELD score ≤6 do not experience any complications. Those who decompensate during pregnancy have a MELD score ≥10 [4]. Our patient had a MELD score of 14 and a Child-Pugh score of B. The Child-Pugh score is a clinical tool used to assess the severity of liver dysfunction, especially in patients with cirrhosis. It is based on five parameters. A Child-Pugh score of B indicates moderately decompensated liver disease, where the liver is significantly impaired but not in the most severe state [7].

Additionally, it is crucial to recognize that liver cirrhosis can cause a variety of complications during pregnancy, including hepatic decompensation, variceal hemorrhage, and impaired fetal growth. In pregnant women with cirrhosis, the most common and serious complication is variceal bleeding. Due to worsening of portal hypertension owing to increased circulating blood volume and direct pressure of the gravid uterus on the inferior vena cava, impairing venous return, variceal bleeding occurs mainly during the second trimester and in the second stage of labor [3].

High mortality and morbidity rates are associated with upper gastrointestinal bleeding from varices in women who already have cirrhosis and portal hypertension, which might show up as ascites, coagulopathy, hepatic encephalopathy, and jaundice. In contrast, women with non-cirrhotic portal hypertension usually have preserved synthetic liver function, and the reproductive system is rarely affected [3]. Our patient underwent an EGD due to a history of esophageal varices identified the previous year. The EGD showed no evidence of esophageal or gastric varices, no fundal varices, and no signs of portal hypertensive gastropathy. A few gastric erosions were observed, but no ulcers were present.

The indications for screening for esophageal varices appear to have at least a moderate indication due to the risks of variceal bleeding, increased mortality, and the opportunity to intervene if varices are identified preemptively. The benefits outweigh the risks, and screening should be done in the second trimester, after organogenesis is complete in the first trimester, and before the greatest risk of bleeding at delivery [8].

Abnormal hematological values are typical in cirrhosis patients. Thrombocytopenia is a frequent liver disease complication. This can adversely affect the treatment, delay surgical/diagnostic procedures, and increase the risk of bleeding. Our patient had thrombocytopenia of 85.3 × 10^9^/L secondary to cirrhosis. The patient had coagulopathy of liver disease (prothrombin time of 17.2 seconds, international normalized ratio of 1.28, and partial thromboplastin time of 46.2 seconds), and she had hypersplenism with no intervention required. Thrombocytopenia is seen in about 70% of women with liver cirrhosis. Multiple factors can contribute to the development of thrombocytopenia in cirrhotic patients. The major mechanisms for thrombocytopenia in liver cirrhosis are platelet sequestration in the spleen and decreased production of thrombopoietin in the liver [9].

From a fetal perspective, pregnancy in women with underlying cirrhosis has been associated with an increase in prematurity, spontaneous abortions, and maternal-fetal mortality. The potential for intrauterine growth restriction necessitates thorough prenatal care. [8] Prematurity has been observed in up to two-thirds of cases, although the majority of these infants are born from 30 weeks onwards. Delivery at <30 weeks may occur in one in five pregnancies. It is associated with higher MELD scores at conception, but not with the etiology of liver disease [4]. Non-cirrhotic portal hypertension has been reported to have better outcomes than cirrhotic portal hypertension [8].

The incidence of spontaneous abortion, which refers to pregnancy loss occurring before 24 weeks of gestation, is similar to that of the general population. There is no significant increased risk of stillbirth, miscarriage, or congenital malformation [4]. Regular ultrasound evaluations are crucial to monitor fetal development and ensure timely interventions if complications arise.

Traditionally, vaginal delivery with a short second stage of labor is often recommended alongside forceps or vacuum extraction, if needed. However, prolonged vaginal delivery may increase variceal bleeding due to repeated Valsalva maneuvers, leading to an interest in considering cesarean sections as an alternative. Cesarean sections may be required for obstetric indications but carry an increased risk of bleeding complications, especially in cases of portal hypertension. There is no data comparing vaginal delivery and cesarean sections, but vascular surgery support is advised for difficult-to-control collateral bleeding [8].

The patient’s condition required a multidisciplinary team approach for optimal management. The multidisciplinary team agreed that there was no contraindication to proceed with normal vaginal delivery, with close observation for decompensation signs. The cardiology team ruled out cardiac causes for her lower limb edema. The nephrology team noted that the albumin-to-creatinine ratio was within the nephrotic range. The hematology team assessed her thrombocytopenia and coagulopathy as secondary complications of her liver disease. The intensive care unit team was consulted for post-delivery observation. The patient proceeded with vaginal delivery assisted by vacuum extraction to cut short the second stage of labour. She delivered a healthy male infant weighing 2.33 kg with Apgar scores of 7 and 10. Active management of the third stage of labor was performed. Subsequently, she developed considerable postpartum hemorrhage with a drop in hemoglobin from 10 g/dL to 6 g/dL, necessitating the transfusion of four units of FFP and six units of platelets. Factor VIIa was administered as per hematology recommendations, aligning with emergency protocols for cirrhosis-related coagulopathy. [3] Postpartum patients are counseled regarding contraceptives and medical nutrition, with a recommendation of oral feeding of a soft, hepatic, low-fat diet. The patient responded well to the management, with comprehensive follow-up care arranged with the hepatology team.

However, this case report is limited by its singular focus on one patient’s experience. The unique factors influencing her compliance and motivation may not apply to other patients, highlighting the need for larger, diverse studies to validate these results. Future research should establish standardized guidelines for managing liver disease during pregnancy, conduct more studies to understand the epidemiology of pregnancy-related liver disease in the Middle East, assess long-term impacts, and investigate the effectiveness of treatment options for optimizing management strategies.

## Conclusions

This case of a young female with liver cirrhosis and portal hypertension demonstrates the importance of multidisciplinary collaboration, including obstetricians, hepatologists, gastroenterologists, nephrologists, hematologists, cardiologists, anesthesiologists, and nutritionists, to develop a tailored plan, considering the timing and mode of delivery, which may vary based on maternal and fetal conditions. As liver disease can significantly impact pregnancy, ongoing research and improved clinical guidelines are essential to enhance care for these patients. There is a critical need for clear, evidence-based guidelines and early referral to specialized tertiary centers, where expert care and resources are available for preconception counseling, follow-up, and effective management of such high-risk pregnancies. By sharing this case, we have contributed to a growing body of evidence that can inform clinical practice, ultimately leading to better health outcomes.
